# Application of Real-Time Quantitative PCR to Detect Mink Circovirus in Naturally and Experimentally Infected Minks

**DOI:** 10.3389/fmicb.2018.00937

**Published:** 2018-05-14

**Authors:** Xingyang Cui, Yunjia Shi, Lili Zhao, Shanshan Gu, Chengwei Wei, Yan Yang, Shanshan Wen, Hongyan Chen, Junwei Ge

**Affiliations:** ^1^College of Veterinary Medicine, Northeast Agricultural University, Harbin, China; ^2^Laboratory Animal and Comparative Medicine Unit, Harbin Veterinary Research Institute, Chinese Academy of Agricultural Sciences, Harbin, China; ^3^Northeastern Science Inspection Station, China Ministry of Agriculture Key Laboratory of Animal Pathogen Biology, Harbin, China

**Keywords:** mink circovirus, Real-Time quantitative PCR, sensitivity, specificity, repeatability

## Abstract

The mink circovirus (MiCV), a newly discovered pathogen, is associated with diarrhea in farmed minks. The prevalence and economic importance of this virus remain poorly understood, and a quantitative method for diagnosis of MiCV infection has not been established. This research aims to develop a highly specific, sensitive, and quantitative assay for MiCV. A Real-Time quantitative polymerase chain reaction (qPCR) assay was developed to detect different isolates of the MiCV in mink samples. The qPCR system is highly sensitive with a detection limit of as low as 10 viral DNA copies. The specificity of this qPCR assay was supported by the absence of cross-reaction with other pathogens. The coefficients of variation were low for both inter-assay and intra-assay variabilities. In addition, the results also expressed the distribution of MiCV in infectious mink tissues with high levels of virus in the skeletal muscle and heart. The heart occupied a higher proportion than other tissues, which can be considered the primary source of test material. This qPCR method could be a useful tool for epidemiological studies and disease management. This method for MiCV is highly specific, sensitive, repeatable, quantitative, and can rapidly determine viral load levels in different tissues samples.

## Introduction

The mink circovirus (MiCV) as reported initially as a novel pathogen in diarrheal minks in Dalian, China (Lian et al., 2014). MiCV is a member of *Circovirus* belonging to the *Circoviridae* family. MiCV is a small non-enveloped icosahedral virus with a circular single-stranded DNA genome of 1,753 nucleotides (Desselberger, 2002). The genome contains two major open reading frames (ORFs), designated ORF1 (Rep gene) and ORF2 (Cap gene).

Circoviruses have been identified in numerous species associated with various clinical disorders (Decaro et al., 2014; Adams et al., 2017; Breitbart et al., 2017), including lethal diseases and asymptomatic infections (Todd, 2004). Circovirus infections are also associated to immunosuppression and lymphoid depletion, which likely increase the severity of secondary infection (Segalés, 2012). Epidemiological investigations have provided evidence that MiCV is endemic in some mink farms in China (Wang et al., 2015b). However, the pathogenic role of MiCV in single or polymicrobial infections is unclear, and the relationship between MiCV distribution in tissue and pathogenicity is remain unknown. Therefore, to establish a rapid, sensitive and specific method applicable for quantitative analysis of this virus is essential for clinical disease management and epidemiological surveillance.

The fundamental diagnostic method of viral disease based on virus isolation is unavailable because no *in vivo* cell culture system is yet available for the propagation of MiCV. Up to now, only conventional PCR for detecting MiCV has been reported (Wang et al., 2015a). Thus, to develop a more sensitive and quantitative method is necessary for the enhanced detection of MiCV.

A Real-Time quantitative polymerase chain reaction (qPCR) techniques have proven to diagnose important viral livestock diseases and have become established scientific tools in veterinary virology and disease control (Hoffmann et al., 2009; Vázquez et al., 2017). Furthermore, the SYBR Green I-based qPCR is one of the most effective methods in the differential and rapid detection of a variety of viral pathogens (Varga and James, 2006; Martínez et al., 2008). qPCR tests have been successfully detected for the diagnosis of important diseases caused by DNA or RNA viruses, such as porcine circovirus type 2 (PCV2) (Wei et al., 2008), porcine circovirus type 3 (Wang et al., 2017), canine circovirus (DogCV) (Hsu et al., 2016), goose circovirus (Tian et al., 2010), aleutian mink disease virus (AMDV) (Prieto et al., 2014), canine parvovirus type 2 (Wang et al., 2016), and porcine parvovirus (Yang et al., 2016).

In this study, the qPCR method was employed for MiCV DNA quantification. MiCV standard curve was established by routine methods and compared to the results of conventional PCR method. Furthermore, the application of qPCR assays provides a highly sensitive, specific and repeatable screening that are essential for exposure assessments of infectious virus. This study demonstrated the use of qPCR for the detection of viral distribution in different tissues caused by MiCV in clinical infected minks.

## Materials and methods

### Viruses and samples

The MiCV HEB15 strain was isolated previously by our team (GenBank Accession No. KX268345) and used as the positive control for the qPCR and conventional PCR assay. Negative controls to test the specificity of qPCR included AMDV and DogCV isolated by our laboratory from Heilongjiang Province, respectively (GenBank accession No. KY680280 and No. MF797786). The porcine circovirus 1 (PCV1) and PCV2 were obtained from Dr. Yanwu Wei, Harbin Veterinary Research Institute, the Chinese Academy of Agricultural Sciences (Huang et al., 2012; Wang et al., 2015c). The mink calicivirus (MCV), pseudorabies virus (PRV), rabies virus (RV) and canine adenovirus type 2 (CAV2) were obtained from Dr. Yongjun Wen (Yang et al., 2012), Dr. Tongqing An (Ye et al., 2015), Dr. Jinying Ge (Guo et al., 2009), and Dr. Jiang Qian (Yu et al., 2015), respectively. Vaccine strain mink enteritis virus (MEV), canine distemper virus (CDV), were purchased from QiLu Animal Health Products, LTD., Shandong, China.

The 401 minks clinical samples were obtained from 23 farms in Heilongjiang, Jilin, Liaoning, Hebei, Shandong Provinces, in China, from May 2014 to August 2017.

### Primer design

Primer design for MiCV qPCR was based on the published Cap gene sequence of the MiCV strain. Complete genome sequences of eight different MiCV were obtained from GenBank, using Clustal W to find a well-conserved region within the Cap gene. Primers were designed by the Beacon Designer software. All primers were purchased from company (Comate Bioscience, Changchun, China). The primers used were cap2-F: 5′-CGTATTGTCCAGGTTTGTATGAAG-3′, and cap2-L: 5′-TCACAGGCATTCCCGCTAC-3′.

### DNA extraction

DNA samples were extracted from liver, lung, spleen, heart, duodenum, jejunum, ileum, colon, brain, kidney, skeletal muscle, marrow, and mesenteric lymph nodes. Theses tissue samples were collected from mink samples. Generally, 0.1 gram of each tissue specimens was weighed, added into 500 μL of phosphate buffered saline, all the samples were homogenized, freeze thawed three times, then centrifugated. Total genomic DNA was extracted from 200 μL supernatant of tissue homogenates using genomic DNA Kit (Axygen A Corning Brand, Suzhou, China), according to the manufacturer's protocol. After elution in 100 μL elution buffer, DNA was stored at −80°C before use.

### Preparation of standard DNA solutions

The target cap gene of MiCV was amplified from the total viral DNA following a published protocol, using forward primer McapF: 5′-GGATCCATGCCCGTAAGATCGCGAT-3′ and the reverse primer McapR: 5′-GGTACCTTAAGTTTGCTTTGGG-3′ (Wang et al., 2015a). A 684 bp fragment of the cap gene was amplified by PCR from DNA of MiCV strain HEB15. The amplified product was purified from agarose gels using a quick PCR purification kit (Axygen A Corning Brand, Suzhou, China), then cloned into *E. coli* DH5α cells (TransGen Biotech, Beijing, China) using the pMD18-T vector (Takara Biotechnology, Dalian, China). Recombinant plasmids were purified by the plasmid purification kit (Real-Times, Beijing, China) and validated by sequencing company (Comate Bioscience, Changchun, China). The target plasmid pMDT-Cap with the original concentration was identified by using a Nanodrop 2000 spectrophotometer (Thermo Scientific, Wilmington, DE). The serial tenfold dilutions of the standard plasmid templates were used for the quantitative analysis and the copy number of plasmid was computed referred to the previous study (Luo et al., 2013).

### Establishment of the standard curve for qPCR

Serial 10-fold dilutions containing copies of 3.5 × 10^8^–3.5 × 10^1^ were used as templates to prepare the standard curves. Real-time PCR assays were carried out in triplicate in a 20 μL final volume that included 2 μL of pMDT-Cap plasmid, 4 μL 5 × Golden HS SYBR Green qPCR Mix (Haigene, Harbin, China), 0.4 μL 50 × ROX Reference Dye, 0.8 μL of each primer (10 μM), and 12 μL of nuclease-free water. The qPCR reaction was conducted in 8-tube strip using a ABI Prism 7500 thermocycler (Applied Biosystems, USA) under the following conditions: 95°C for 15 min, then 40 cycles of denaturation for 10 s at 95°C, annealing for 35 s at 55°C and elongation for 45 s at 72°C. A melting curve analysis was performed at the end of the amplification. The dilutions were tested in triplicate and used as quantification standards to construct the standard curve. by plotting the plasmid copy number logarithm against the measured cycle threshold (*Ct*) values. The standard curve, correlation coefficient of the standard curve, and efficiencies were calculated automatically by the LightCycler software. The amount of DNA quantified for each sample was expressed as number of copies/reaction.

### Specificity, sensitivity, and reproducibility assay

Ten viruses that may cause potential cross-reactions in qPCR assay for MiCV were used to evaluate the specificity in this study. The MiCV HEB15 strain was used as the positive control and sterile water was used as the negative control.

To compare the sensitivity of qPCR with conventional PCR, 10-serial pMDT-Cap plasmid dilutions (10^8^–10^1^ copies/μL) were used as templates. The conventional PCR reaction was performed using the protocol described above with the primer pair McapF and McapR.

The repeatability was determined using four different DNA concentrations of viral agent tested. Concentrations of the DNA standard 10^2^, 10^3^, 10^5^, and 10^7^ copies/μL were tested and analyzed by qPCR. For intra-assay variability, each dilution was analyzed in triplicate. To evaluate the inter-assay precision of the assay, each dilution was analyzed in different runs performed by two different laboratory technicians on different days. The coefcient of variation (CV) was determined following the formula: *CV* = (SD [*Ct*-value]/overall mean [*Ct*-value]) × 100.

### Detection in samples from infected minks

A total of 401 clinical samples including 52 serum sample, 319 different tissues of minks and 30 healthy mink fecal samples were collected from 5 provinces, including Heilongjiang, Jilin, Shandong, Hebei, and Liaoning in China in 2014–2017. These samples were examined in terms of presence of MiCV infection in qPCR and were assayed in duplicate. Furthermore, these samples were also detected using conventional PCR simultaneously.

There are 8 artificial infection minks, 22 naturally infected minks and the 3 healthy control minks were collected to characterize the tissue distribution of MiCV in minks. The tissues, including liver, lung, spleen, heart, duodenum, jejunum, ileum, colon, brain, kidney, skeletal muscle, marrow, and mesenteric lymph nodes, were detected by qPCR to evaluate viral load. The viral load was quantified by qPCR using 2 μL of DNA per reaction. The DNA copy number of each sample was converted to copy number per gram by using the calculated *Ct*-value determined from the standard curve. All the samples were tested in triplicates.

## Results

### Establishment of the standard curve for qPCR

**Figure 1:**
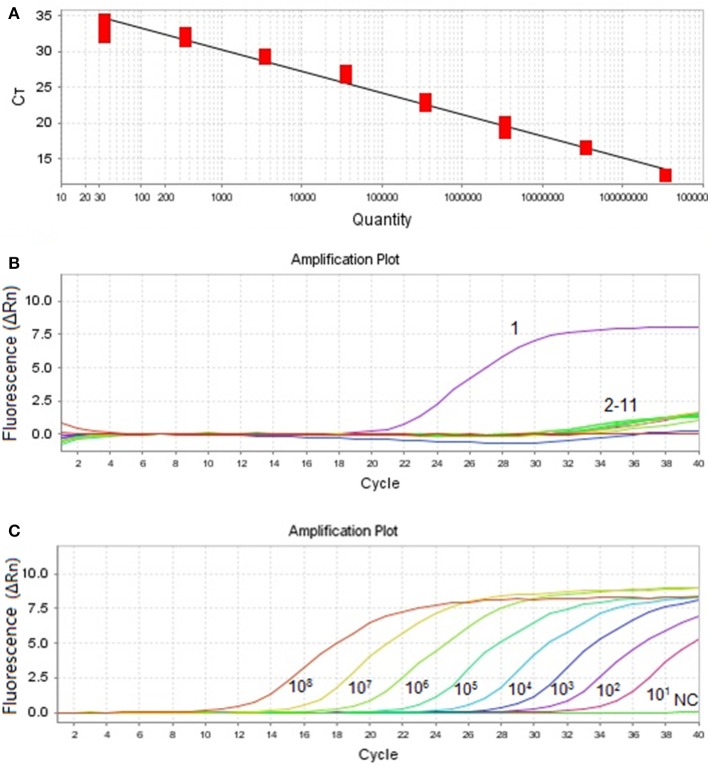
Amplification curve and standard curve of MiCV. **(A)**: Standard curve (Slope: −3.155, Y-Intercept: 39.202, and Efficiency: 107.447) was analyzed with the ABI7500 software. **(B)**: Specificity of the qPCR. Ten other viral pathogens were used for the specificity test. 1: MiCV HEB15 strain DNA; 2–11: DNA and RNA samples of AMDV, MEV, CDV, PRV, MCV, CAV2, RV, PCV1, PCV2, DogCV, and H_2_O. **(C)**: Amplification curves. Ten-fold dilutions of standard DNA ranging from 10^8^ copies/μL to 10^1^ copies/μL were used as standard controls.

As shown in Figure 1A, the qPCR amplification curves were generated by using 10-fold dilutions of plasmid PMDT-Cap template with the cap2-F and cap2-L as primers. The MiCV assay covered a linear range 8 orders of magnitude from 3.5 × 10^8^ copies/μL to 3.5 × 10^1^ copies/μL. *Ct*-values were plotted against the known copy numbers of the standard controls. Thus, a correlation coefficient (R^2^) of 0.974, a slope of −3.155, an efficiency of 107.447% and the Y intercept 39.202 were obtained in this study and the following formula: *Y* = −3.155X+39.202 (Y = threshold cycle, X = natural log of concentration (copies/μL) was achieved.

### Specificity, sensitivity, and reproducibility of qPCR detection

In this assay, plasmid pMDT-Cap was used as DNA template of MiCV. The results indicated that only detected MiCV gave amplification products. Under the same conditions, AMDV, DogCV, PCV1, PCV2, MCV, PRV, RV, CAV2, CDV, and MEV did not provide positive reactions (Figure 1B). The sensitivity assay was demonstrated and compared with that of conventional PCR. (Figure 1C). The detection limit of the MiCV qPCR was 10^1^ copies, while the conventional PCR, 1 × 10^6^ copies (Wang et al., 2015a). This indicates that the qPCR assay for MiCV was 10^5^ times more sensitive than that of the conventional PCR. The qPCR assay expressed a high repeatability with coefficient of variation (CV) within runs (intra-assay variability) and between runs (inter-assay variability) that ranged from 0.73 to 1.69% and 0.97 to 2.18%, respectively (Table 1). Our results, therefore, showed that the qPCR assay is highly sensitive, specific and reproducible.

**Table 1:** Intra- and inter-assay reproducibility of qPCR.

**DNA standard (copies/μL)**	**Intra-assay reproducibility**		**Inter-assay reproducibility**	
	**Mean *C*t ± SD**	**CV (%)**	**Mean *C*t ± SD**	**CV (%)**
10^7^	16.07 ± 0.25	1.58	16.53 ± 0.21	1.29
10^5^	22.48 ± 0.38	1.69	22.92 ± 0.50	2.18
10^3^	28.98 ± 0.21	0.73	29.28 ± 0.28	0.97
10^2^	31.33 ± 0.25	0.79	31.82 ± 0.63	1.98

### Application of real-time PCR assays on clinical infected minks

The application of qPCR assays on clinically infected minks was evaluated through the 401 clinical samples. The positive rates of MiCV in different provinces were 30.30% in Heilongjiang Province, 52.88% in Shandong Province, 67.90% in Hebei Province, 38.46% in Jilin Province, and 58.46% in Liaoning Province (Table 2). Conventional PCR was also performed on the same samples. However, the positivity rates of MiCV infection determined using conventional PCR were 18.18, 30.77, 37.04, 28.85, and 27.69% in Heilongjiang, Shandong, Hebei, Jilin, and Liaoning Province, respectively (Table 2). Among those that were positive by qPCR (401 samples), 113 samples were also positive by conventional PCR detection. Another 203 samples were determined as negative by either qPCR or conventional PCR virus. These results showed that the qPCR screen was more sensitive than conventional PCR.

**Table 2:** Detection of MiCV in minks from different provinces.

		**Convention PCR**		**qPCR**	
**Province**	**Farm**	**Number positive/number tested**	**Positive rate (%)**	**Number positive/number tested**	**Positive rate (%)**
Heilongjiang	1	5/21	23.81	7/21	33.33
	2	0/16	0.00	3/16	18.75
	3	6/18	33.33	8/18	44.44
	4	3/16	18.75	4/16	25.00
	5	4/28	14.29	8/28	28.57
Shandong	1	8/19	42.11	10/19	52.63
	2	6/16	37.50	10/16	62.50
	3	5/18	27.78	8/18	44.44
	4	4/20	20.00	9/20	45.00
	5	9/31	29.03	18/31	58.06
Hebei	1	10/24	41.67	17/24	70.83
	2	5/17	29.41	11/17	64.71
	3	4/12	33.33	8/12	66.67
	4	5/13	38.46	9/13	69.23
	5	6/15	40.00	10/15	66.67
Liaoning	1	6/23	26.09	11/23	47.83
	2	5/16	31.25	10/16	62.50
	3	3/15	20.00	9/15	60.00
	4	4/11	36.36	8/11	72.73
Jilin	1	6/13	46.15	7/13	53.85
	2	4/14	28.57	5/14	35.71
	3	3/13	23.08	4/13	30.77
	4	2/12	16.67	4/12	33.33
Total		113/401	28.18	198/401	49.38

### Quantification of MiCV viral DNA from different tissues

To investigate MiCV distribution in infected minks, 8 artificially infected minks, 22 naturally infected minks, and 3 control minks were used and determined by the qPCR. All of the 8 artificially infected minks were MiCV positive, and MiCV can be detected in all these 13 tissues. The viral loads of 8 artificially infected minks are shown in Table 3. The results showed that artificially infected minks had the highest level of mean viral loads in heart (6.93 × 10^8^ copies/g), followed by skeletal muscle (4.75 × 10^8^ copies/g), brain (1.36 × 10^8^ copies/g), duodenum (1.36 × 10^8^ copies/g), kidney (6.16 × 10^7^ copies/g), jejunum (2.44 × 10^7^ copies/g), lung (1.82 × 10^7^ copies/g), mesenteric lymph nodes (1.54 × 10^7^ copies/g), colon (1.32 × 10^7^ copies/g), spleen (1.28 × 10^7^ copies/g), liver (1.24 × 10^7^ copies/g), and lastly, marrow (9.27 × 10^6^ copies/g), and ileum (8.80 × 10^6^ copies/g).

**Table 3:** Quantities of MiCV DNA in the tissues of artificially-infected minks by qPCR.

	**Virus load in infected minks (copies/g)**							
**Tissues**	**No. 1**	**No. 2**	**No. 3**	**No. 4**	**No. 5**	**No. 6**	**No. 7**	**No. 8**
liver	2.92 × 10^7^	5.82 × 10^5^	1.60 × 10^7^	3.79 × 10^5^	3.87 × 10^7^	1.06 × 10^7^	1.06 × 10^6^	2.47 × 10^6^
lung	5.52 × 10^6^	2.65 × 10^7^	2.46 × 10^7^	1.06 × 10^7^	1.05 × 10^7^	6.63 × 10^6^	2.35 × 10^7^	3.80 × 10^7^
spleen	2.35 × 10^7^	4.73 × 10^6^	2.79 × 10^7^	1.04 × 10^6^	1.21 × 10^7^	3.14 × 10^7^	4.52 × 10^5^	1.05 × 10^6^
heart	1.23 × 10^8^	3.08 × 10^8^	4.81 × 10^7^	3.6 × 10^8^	6.93 × 10^7^	1.26 × 10^9^	6.27 × 10^7^	3.32 × 10^9^
duodenum	1.40 × 10^7^	2.43 × 10^6^	2.32 × 10^7^	4.91 × 10^7^	7.27 × 10^6^	8.22 × 10^8^	1.64 × 10^8^	2.37 × 10^6^
jejunum	3.36 × 10^7^	7.60 × 10^5^	3.36 × 10^7^	2.06 × 10^6^	1.60 × 10^7^	9.16 × 10^6^	9.23 × 10^7^	7.77 × 10^6^
ileum	4.61 × 10^6^	7.59 × 10^6^	2.02 × 10^7^	1.33 × 10^7^	1.14 × 10^7^	4.36 × 10^6^	8.65 × 10^6^	2.59 × 10^5^
colon	1.45 × 10^7^	1.16 × 10^6^	1.89 × 10^7^	1.66 × 10^6^	3.95 × 10^6^	6.44 × 10^7^	3.98 × 10^5^	3.05 × 10^5^
brain	1.63 × 10^7^	1.78 × 10^7^	1.86 × 10^7^	5.11 × 10^8^	2.32 × 10^8^	1.74 × 10^9^	3.51 × 10^7^	2.97 × 10^7^
kidney	5.08 × 10^7^	1.22 × 10^7^	1.96 × 10^6^	2.53 × 10^8^	4.61 × 10^6^	4.59 × 10^7^	1.86 × 10^6^	1.23 × 10^8^
skeletal muscle	1.49 × 10^7^	9.63 × 10^7^	1.04 × 10^8^	2.14 × 10^9^	5.08 × 10^7^	1.48 × 10^8^	1.48 × 10^8^	1.10 × 10^9^
marrow	1.29 × 10^7^	2.41 × 10^5^	1.83 × 10^6^	3.11 × 10^6^	1.98 × 10^7^	1.58 × 10^7^	1.66 × 10^7^	3.95 × 10^6^
mesenteric lymph nodes	2.38 × 10^7^	3.56 × 10^6^	6.25 × 10^7^	7.64 × 10^6^	6.18 × 10^6^	3.83 × 10^6^	8.12 × 10^6^	7.24 × 10^6^

Moreover, the number of MiCV DNA copies in different tissues of 22 naturally infected minks was shown below: heart (3.35 × 10^9^–4.95 × 10^6^ copies/g), skeletal muscle (4.89 × 10^9^–9.49 × 10^5^ copies/g), brain (1.75 × 10^9^–3.50 × 10^6^ copies/g), kidney (2.11 × 10^9^–1.53 × 10^6^ copies/g), duodenum (8.23 × 10^8^–2.33 × 10^6^ copies/g), jejunum (3.75 × 10^8^–7.23 × 10^5^ copies/g), lung (1.23 × 10^8^–5.16 × 10^6^ copies/g), liver (1.64 × 10^8^–5.80 × 10^5^ copies/g), colon (7.89 × 10^7^–2.38 × 10^5^ copies/g), mesenteric lymph nodes (6.64 × 10^7^–3.05 × 10^5^ copies/g), spleen (3.16 × 10^7^–3.48 × 10^5^ copies/g), marrow (2.03 × 10^7^–2.09 × 10^4^ copies/g), and ileum (3.18 × 10^7^–2.51 × 10^4^ copies/g).

In a descending order, the mean viral loads per tissue were as follows: skeletal muscle (5.31 × 10^8^ copies/g), heart (5.02 × 10^8^ copies/g), kidney (2.94 × 10^8^ copies/g), brain (2.45 × 10^8^ copies/g), and duodenum (1.31 × 10^8^ copies/g) samples contained the highest viral loads, whereas jejunum (5.11 × 10^7^ copies/g), lung (4.68 × 10^7^ copies/g), colon (2.68 × 10^7^ copies/g), mesenteric lymph nodes (2.59 × 10^7^ copies/g), liver (1.88 × 10^7^ copies/g), marrow (1.44 × 10^7^ copies/g), spleen (1.04 × 10^7^ copies/g), and ileum (9.58 × 10^6^ copies/g) had the lowest viral loads. Meanwhile, no MiCV was detected in the 3 healthy control minks. In general, the viral load between the naturally and experimentally infected minks in the different tissues were similar.

In addition, these tissues were also used to investigate MiCV distribution in infected minks. The percentages of samples, which tested positive for virus from minks, were as follows: in lung 28/30 (93.33%), in heart 28/30 (93.33%), in duodenum 26/30 (86.67%), in jejunum 24/30 (80.00%), in skeletal muscle 24/30 (80.00%), in colon 22/30 (73.33%), in kidney 22/30 (73.33%), in ileum 18/30 (60.00%), in marrow 16/30 (53.33%), and in mesenteric lymph nodes 16/30 (53.33%). Furthermore, all the infected minks had detectable virus in the liver, spleen, and brain.

## Discussion

MiCV is a newly novel pathogen in diarrheal minks in China (Lian et al., 2014). Until now, the pathogenic role of MiCV is unknown, and the prevalence and economic importance of this virus are not fully understood. The conventional PCR method had lower sensitivity, and there are no accepted methods of standard culture to quantify viruses in different tissues of infectious minks. qPCR is used as a diagnostic tool because it is more rapid, sensitive, and allowing quantitative. Thus, qPCR is a useful tool in the study of the epidemiology of viral diseases (He et al., 2016), which makes it necessary for MiCV detection. In this study, a pair of primers targeting the capsid protein gene segment of MiCV was designed and the reaction conditions for qPCR were optimized through repeated experiments. At first, we chose rep gene to develop the qPCR assay, but failed to construct recombinant rep gene plasmid. For some unknown reason, rep gene or part of the rep gene fragment can't be cloned into competent cells using the pMD18-T vector. We speculated that the rep gene may be toxic and damaging cells. So we selected a well-conserved region within the Cap gene to design the primers. Taking into account its possible variation of the cap gene in the future, we should note to pay attention to that gene changes may affect the detection results when using this method.

According to previous study, when *Ct* is plotted against a template DNA concentration, a regression analysis of the data should give an optimal *R*^2^-value close to 1, while the efficiency of PCR should be between 90 and 110% (Rasmussen, 2001; Tichopad et al., 2003; Hughes et al., 2007). Figure 1A demonstrated the fair correlation between copy number and *Ct*-value (a slope of −3.155, an efficiency of 107.447%, and the Y intercept 39.202), in which it indicated that this method is highly efficient, and may prove a suitable approach for MiCV diagnosis.

The results of the specificity tests indicated that the qPCR assay can successfully detect the MiCV in infected minks, and showed no cross-reaction with other viral pathogens, thereby ensuring high accuracy and reliability of the assay in MiCV detection. With the developed qPCR assay, we compared the performance of this novel technique with conventional PCR in detecting MiCV. Based on serially diluted DNA standards, the detection limit of the MiCV qPCR assay was as low as 10 viral DNA copies, which had 10^5^-fold higher sensitivity than that of conventional PCR (Wang et al., 2015a). The qPCR assay has been applied to detect clinic samples, where 32 infected samples were identified, while only 23 samples were detected by the conventional PCR and control group were all negative (Table 4). qPCR not only offered a higher sensitivity to qPCR assay, but also avoided the post PCR processing. In addition, the qPCR assay permitted the simultaneous detection and quantification of DNA, and it also provided a more objective final analysis. The reproducibility of the qPCR method was determined by calculating the CV for each set of triplicate replicas based on copy number (between runs) (Bustin et al., 2009; León et al., 2017). In this study, the CV for replicates between runs ranged from 0.73 to 2.18%, which showed high reproducibility with intra-assay and inter-assay variabilities.

**Table 4:** Comparison of the results obtained from 35 selected samples using conventional PCR and qPCR.

**Samples No**.	**qPCR^a^ (MiCV copies/g DNA)**	**Conventional PCR**
**INFECTED MINKS**		
1	1.64 × 10^8^	+
2	8.23 × 10^5^	−
3	3.09 × 10^8^	+
4	1.26 × 10^5^	−
5	3.50 × 10^6^	+
6	3.05 × 10^6^	+
7	4.88 × 10^5^	−
8	1.10 × 10^6^	+
9	6.18 × 10^5^	−
10	3.31 × 10^9^	+
11	3.36 × 10^7^	+
12	3.75 × 10^8^	+
13	2.33 × 10^8^	+
14	1.29 × 10^9^	+
15	1.60 × 10^7^	+
16	1.14 × 10^7^	+
17	1.75 × 10^5^	−
18	2.14 × 10^9^	+
19	2.98 × 10^7^	+
20	5.76 × 10^5^	−
21	1.59 × 10^8^	+
22	5.11 × 10^5^	−
23	1.48 × 10^8^	+
24	7.28 × 10^6^	+
25	2.53 × 10^8^	+
26	4.51 × 10^5^	−
27	2.59 × 10^5^	−
28	3.60 × 10^8^	+
29	6.53 × 10^7^	+
30	5.33 × 10^7^	+
31	4.96 × 10^8^	+
32	6.28 × 10^7^	+
**UNINFECTED MINKS**		
1	0	−
2	0	−
3	0	−

*(+) detected as positive by conventional PCR*.

*(−) detected as negative by conventional PCR*.

a *Means of triplicate copy number*.

The total positive rate of MiCV in different farms 49.38% had some differences in the prevalence of the MiCV infection in different provinces. According to Wang et al. they obtained the positive rate was 54.6% (Wang et al., 2015a).

The qPCR method was able to quantify the level of viral DNA in different tissues of the MiCV infected minks. In the present study, viral DNA loads of MiCV infected minks were found to be widely distributed in various tissues of artificial infection and naturally infection samples. The naturally infected minks mean viral loads of skeletal muscle, heart, kidney and brain was present at higher levels, 5.31 × 10^8^ copies/g, 5.02 × 10^8^ copies/g, 2.94 × 10^8^ copies/g, and 2.45 × 10^8^ copies/g, respectively. Among artificial infection minks, the order of tissues with higher mean viral loads is heart (6.93 × 10^8^ copies/g), skeletal muscle (4.75 × 10^8^ copies/g), brain (1.36 × 10^8^ copies/g), then duodenum (1.36 × 10^8^ copies/g). However, the viral DNA loads in lymphoid tissues are not very high. The heart has a higher mean viral loads in both naturally infected minks and experimentally infected minks. We also found that the heart can be tested positive for MiCV easily, due to its relatively high testing rate in infected minks. However, other tissues can't satisfy these two requirements simultaneous. Based on these reasons, the heart can be considered the first choice of tissue for detecting MiCV in infected minks and might be a key index for MiCV detection.

Furthermore, the pathogenic role of MiCV is still unclear, though the virus distribution in tissues may be related with the pathogenicity. Hence, the result of the viral loads in different tissue samples of MiCV-infected minks may be conducive to MiCV pathogenicity-related research in the future.

The qPCR assays for PCV2 and DogCV detection have been previously reported (Wei et al., 2008; Hsu et al., 2016), but the sequence of these viruses was different. According to these references, we try to explore an qPCR assay to detect MiCV. To our knowledge, this is the first study to apply the use of qPCR technology in a diagnostic test for the distribution of MiCV in the tissues of infected minks. To sum up, heart tissues in MiCV-infected minks showed a higher viral loads, compared with other tissues examined. qPCR method, which is rapid, specific, sensitive, repeatable, and quantitative, made this finding possible. This finding but also provides further insights into detection of MiCV for clinically infected minks and for the diagnosis of viruses where heart tissues are the primary source of test material.

A highly sensitive, specific, repeatable and quantitative MiCV qPCR method for the detection of infected mink samples has been developed and established in this study. Our result also showed that the heart is maybe a priority source of test material. This technique has the potential to be applied in the experimental infections and clinic samples, which may help to control the spread of the disease.

## Ethics statement

This study was performed in accordance with the recommendations in the Guide for the Care and Use of Laboratory Animals of the Ministry of Health, China. Prior to experiments, the protocol of the current study was reviewed and approved by the Institutional Animal Care and Use Committee of Northeast Agricultural University (approved protocol number 2014-SRM-36). Samples were collected only from animals for laboratory analyses, avoiding unnecessary pain and suffering of the animals. The owners gave their written consent for sample collection, and the locations where we sampled are not privately owned or protected in any way. The studies did not involve endangered or protected species.

## Author contributions

JG and HC: Conceived and designed the experiments; XC, JG, YS, SW, and LZ: Performed the experiments; JG, XC, and LZ: Analyzed the data; LZ, SG, CW, and YY: Contributed reagents, materials, and analysis tools; JG and XC: Wrote the paper. All authors read and approved the final manuscript.

### Conflict of interest statement

The authors declare that the research was conducted in the absence of any commercial or financial relationships that could be construed as a potential conflict of interest.

## References

[B1] Adams M. J. Lefkowitz E. J. King A. M. Q. Harrach B. Harrison R. L. Knowles N. J.. (2017). Changes to taxonomy and the international code of virus classification and nomenclature ratified by the international committee on taxonomy of viruses (2017). Arch. Virol. 162, 2505–2538. 10.1007/s00705-017-3358-5 28434098

[B2] Breitbart M. Delwart E. Rosario K. Segalés J. Varsani A. Consortium I. R. (2017). ICTV virus taxonomy profile: circoviridae. J. Gen. Virol. 98, 1997–1998. 10.1099/jgv.0.000871 28786778 PMC5656780

[B3] Bustin S. A. Benes V. Garson J. A. Hellemans J. Huggett J. Kubista M.. (2009). The MIQE guidelines: minimum information for publication of quantitative real-time PCR experiments. Clin. Chem. 55, 611–622. 10.1373/clinchem.2008.112797 19246619

[B4] Decaro N. Martella V. Desario C. Lanave G. Circella E. Cavalli A.. (2014). Genomic characterization of a circovirus associated with fatal hemorrhagic enteritis in dog, Italy. PLoS ONE 9:e105909. 10.1371/journal.pone.0105909 25147946 PMC4141843

[B5] Desselberger U. (2002). Virus Taxonomy: Classification and Nomenclature of Viruses, in Seventh Report of the International Committee on Taxonomy of Viruses, eds van Regenmortel M. H. V. Fauquet C. M. Bishop D. H. L. Carstens E. B. Estes M. K. Lemon S. M. Maniloff J. Mayo M. A. (San Diego, CA: Academic Press), 221–222.

[B6] Guo L. Feng N. Yang S. Wang X. Ge J. Xia X.. (2009). [Reverse genetic system for rabies virus vaccine Evelyn-Rokitnicki-Abelseth strain]. Acta Microbiol. Sin. 49, 949–954. 19873761

[B7] He Y. Yan H. Hua W. Huang Y. Wang Z. (2016). Selection and validation of reference genes for quantitative real-time pcr in *gentiana macrophylla*. Front Plant Sci. 7:945. 10.3389/fpls.2016.00945 27446172 PMC4925707

[B8] Hoffmann B. Beer M. Reid S. M. Mertens P. Oura C. A. van Rijn P. A.. (2009). A review of RT-PCR technologies used in veterinary virology and disease control: sensitive and specific diagnosis of five livestock diseases notifiable to the World Organisation for Animal Health. Vet. Microbiol. 139, 1–23. 10.1016/j.vetmic.2009.04.034 19497689

[B9] Hsu H. S. Lin T. H. Wu H. Y. Lin L. S. Chung C. S. Chiou M. T.. (2016). High detection rate of dog circovirus in diarrheal dogs. BMC Vet. Res. 12:116. 10.1186/s12917-016-0722-8 27315792 PMC4912760

[B10] Huang L. Lu Y. Wei Y. Guo L. Liu C. (2012). Identification of three new type-specific antigen epitopes in the capsid protein of porcine circovirus type 1. Arch. Virol. 157, 1339–1344. 10.1007/s00705-012-1268-0 22437253

[B11] Hughes S. Weksberg R. Moldovan L. Squire J. A. Hughes S. Weksberg R. (2007). Use of quantitative PCR for the detection of genomic microdeletions or microduplications. PCR: Methods Exp. 1, 49–62.

[B12] León C. M. Muñoz M. Hernández C. Ayala M. S. Flórez C. Teherán A.. (2017). Analytical performance of four polymerase chain reaction (PCR) and real time PCR (qPCR) assays for the detection of sixleishmania species DNA in Colombia. Front. Microbiol. 8:1907. 10.3389/fmicb.2017.01907 29046670 PMC5632848

[B13] Lian H. Liu Y. Li N. Wang Y. Zhang S. Hu R. (2014). Novel circovirus from mink, China. Emerg. Infect. Dis. 20, 1548–1550. 10.3201/eid2009.140015 25148585 PMC4178405

[B14] Luo J. G. Ge J. W. Tang L. J. Qiao X. Y. Jiang Y. P. Cui W.. (2013). Development of a loop-mediated isothermal amplification assay for rapid detection of bovine parvovirus. J. Virol. Methods 191, 155–161. 10.1016/j.jviromet.2012.05.002 22584269

[B15] Martínez E. Riera P. Sitjà M. Fang Y. Oliveira S. Maldonado J. (2008). Simultaneous detection and genotyping of porcine reproductive and respiratory syndrome virus (PRRSV) by real-time RT-PCR and amplicon melting curve analysis using SYBR Green. Res. Vet. Sci. 85, 184–193. 10.1016/j.rvsc.2007.10.003 18054369

[B16] Prieto A. Díaz-Cao J. M. Fernández-Antonio R. Panadero R. Díaz P. López C.. (2014). Application of real-time PCR to detect Aleutian Mink Disease Virus on environmental farm sources. Vet. Microbiol. 173, 355–359. 10.1016/j.vetmic.2014.07.024 25183237

[B17] Rasmussen R. (2001). Quantification on the LightCycler, in Rapid Cycle Real-Time PCR: Methods and Applications, (Berlin; Heidelberg: Springer), 21–34.

[B18] Segalés J. (2012). Porcine circovirus type 2 (PCV2) infections: clinical signs, pathology and laboratory diagnosis. Virus Res. 164, 10–19. 10.1016/j.virusres.2011.10.007 22056845

[B19] Tian J. Guo J. Sun H. X. Yuan H. X. Chen W. H. Yu X. P. (2010). Development of a real-time PCR assay for detection of goose circovirus. Chin. J. Prev. Vet. Med. 2859, 867–870. 10.3969/j.issn.1008-0589.2010.11.09

[B20] Tichopad A. Dilger M. Schwarz G. Pfaffl M. W. (2003). Standardized determination of real-time PCR efficiency from a single reaction set-up. Nucleic Acids Res. 31, e122–e122. 10.1093/nar/gng122 14530455 PMC219490

[B21] Todd D. (2004). Avian circovirus diseases: lessons for the study of PMWS. Vet. Microbiol. 98, 169–174. 10.1016/j.vetmic.2003.10.010 14741130

[B22] Varga A. James D. (2006). Real-time RT-PCR and SYBR Green I melting curve analysis for the identification of Plum pox virus strains C, EA, and W: effect of amplicon size, melt rate, and dye translocation. J. Virol. Methods 132, 146–153. 10.1016/j.jviromet.2005.10.004 16293321

[B23] Vázquez L. Guadamuro L. Giganto F. Mayo B. Flórez A. B. (2017). Development and use of a Real-Time Quantitative PCR Method for detecting and quantifying Equol-Producing bacteria in human faecal samples and slurry cultures. Front. Microbiol. 8:1155. 10.3389/fmicb.2017.01155 28713336 PMC5491606

[B24] Wang J. Liu L. Li R. Wang J. Fu Q. Yuan W. (2016). Rapid and sensitive detection of canine parvovirus type 2 by recombinase polymerase amplification. Arch. Virol. 161, 1015–1018. 10.1007/s00705-015-2738-y 26729477 PMC7087227

[B25] Wang J. Zhang Y. Wang J. Liu L. Pang X. Yuan W. (2017). Development of a TaqMan-based real-time PCR assay for the specific detection of porcine circovirus3. J. Virol. Methods 248, 177–180. 10.1016/j.jviromet.2017.07.007 28743583

[B26] Wang Y. Liu Y. Lian H. Li N. Zhang L. Hu R. (2015a). A PCR method for detection of mink circivirus. Chin. J. Vet. Sci. 35, 1774–1776. 10.16303/j.cnki.1005-4545.2015.11.09

[B27] Wang Y. Liu Y. Lian H. Li N. Zhang L. Hu R. (2015b). Molecular Epidemiology of Mink Circovirus. Chin. J. Vet. Med. 51, 6–8.

[B28] Wang Y. Lu Y. Dan L. Wei Y. Guo L. Wu H. (2015c). Enhanced Th1-biased immune efficacy of porcine circovirus type 2 Cap-protein-based subunit vaccine when coadministered with recombinant porcine IL-2 or GM-CSF in mice. Appl. Microbiol. Biotechnol. 99, 1155–1163. 10.1007/s00253-014-6167-8 25487886

[B29] Wei Y. W. Liu C. M. Yuan J. Huang L. P. Zhang Z. X. (2008). Detection of porcine circovirus type 2 distributions in the infected pigs by real-time quantitative PCR. Chin. J. Prev. Vet. Med. 30, 924–928.

[B30] Yang B. Wang F. Zhang S. Xu G. Wen Y. Li J.. (2012). Complete genome sequence of a mink calicivirus in China. J. Virol. 86, 13835–13835. 10.1128/JVI.02582-12 23166245 PMC3503044

[B31] Yang Y. Qin X. Zhang W. Li Y. Zhang Z. (2016). Rapid and specific detection of porcine parvovirus by isothermal recombinase polymerase amplification assays. Mol. Cell. Probes 30, 300–305. 10.1016/j.mcp.2016.08.011 27593155

[B32] Ye C. Zhang Q. Z. Tian Z. J. Zheng H. Zhao K. Liu F.. (2015). Genomic characterization of emergent pseudorabies virus in China reveals marked sequence divergence: evidence for the existence of two major genotypes. Virology 483, 32–43. 10.1016/j.virol.2015.04.013 25965793

[B33] Yu Z. Jiang Q. Liu J. Guo D. Quan C. Li B.. (2015). A simplified system for generating recombinant E3-deleted canine adenovirus-2. Plasmid 77, 1–6. 10.1016/j.plasmid.2014.10.005 25450764

